# An Act to Regulate the Practice of Dentistry within the Territory of New Mexico

**Published:** 1893-07

**Authors:** D. W. Manley

**Affiliations:** Secretary, Santa Fe, New Mexico.


Article V.

### AN ACT TO REGULATE THE PRACTICE OF DENTISTRY WITHIN THE TERRITORY OF NEW MEXICO.

*Be it Enacted by the Legislative Assembly of the Territory of New Mexico:*

Section 1. That it shall be unlawful for any person who is not at the time of the passage of this act engaged in the practice of dentistry in the Territory of New Mexico, to commence such practice, unless such person shall have received a certificate from the duly authorized board of dental examiners hereinafter provided for.

Sec. 2. A board of dental examiners, to consist of five practicing dentists within the Territory of New Mexico, is hereby created, whose duty it shall be to carry out the purposes and enforce the provisions of this act. The members of said board shall be appointed by the Governor. The terms for which the members of said board shall hold their offices shall be four years, and until their successors shall be appointed. In case of a vacancy occurring in the membership of said board, such vacancy shall be filled by appointment by the Governor.

SEC. 3. The said board shall, within sixty days after appointment, meet at the capital of the Territory of New Mexico, and organize by electing one of its members president and one secretary thereof. Said board shall meet at least once in each year thereafter, and as often and at such times and places as it may deem proper and necessary. A majority of said board shall at all times constitute a quorum for the transaction of business.

SEC. 4. It shall be the duty of every person who at the time of the passage of this act is engaged in the practice of dentistry in the Territory of New Mexico, within six months from the date of the passage of this act, to cause his or her written application to be filed with the secretary of said board for a certificate to continue in the practice of dentistry within said Territory; and all persons whom the board may find to have been engaged in the practice of dentistry within the Territory of New Mexico for the period of one year next preceeding the passage of this act shall be entitled to receive a certificate from said board of examiners without further examination.

SEC. 5. No person whose name is not registered on the books of said board as a regular practitioner of dentistry, within the time prescribed in the next preceding section, shall be permitted to practice dentistry within the Territory of New Mexico until such person shall have been duly examined by said board and regularly licensed in accordance with the provisions of this act: *Provided, further*, that all persons presenting a diploma from a college recognized as reputable by the National Association of Dental Examiners and paying the sum of five dollars to the secretary of the board shall be entitled to receive a certificate without further examination.

SEC. 6. In order to provide the means for carrying out and enforcing the provisions of this act, said board of examiners shall charge each person applying for a certificate to continue in the practice of dentistry the sum of five dollars for said certificate, and all persons applying for an examination to procure a certificate to commence the practice of dentistry within the Territory of New Mexico shall pay to the secretary of said board, before submitting to said examination, the sum of twenty-five dollars.

SEC. 7. Any person holding a license from said board who shall be charged with immoral or unprofessional conduct may, if found guilty as charged, upon proper investigation had by said board, have his or her license revoked by said board.

SEC. 8. All moneys received by the board shall be held by the secretary thereof as a special fund for paying the necessary expenses and for enforcing the provisions of this act.

The secretary shall give to the board a good and sufficient bond, to be approved by said board, and in an amount to be fixed by the board.

SEC. 9. No part of the salary or other expenses of said board shall be paid out of the Territorial treasury.

SEC. 10. It shall be the duty of the secretary of the board to make an annual report to the Governor of the Territory, at such times as may be directed by the board, and such report shall be signed and approved by the president of the board.

SEC. 11. Any person who shall violate any of the provisions of this act shall be deemed guilty of a misdemeanor, and upon conviction thereof may be fined not less than twenty dollars nor more than one hundred dollars, or be imprisoned in the county jail not less than one month nor more than three months, or by both such fine and imprisonment, in the discretion of the court trying said cause.

SEC. 12. Any justice of the peace of the county in which such violation was committed shall have jurisdiction in all cases of violations of this act, and it shall be the duty of the respective county attorneys to prosecute all violations of this act.

SEC. 13. Nothing in this act shall be construed to interfere with physicians and surgeons in their practice as such.

SEC. 14. This act shall be in force from and after its passage.

D. W. MANLEY, M. D., *Secretary*,
Santa Fe, New Mexico.

---

ARTICLE VI.

